# CXCR4 in Waldenström’s Macroglobulinema: chances and challenges

**DOI:** 10.1038/s41375-020-01102-3

**Published:** 2020-12-03

**Authors:** Lisa Marie Kaiser, Zachary R. Hunter, Steven P. Treon, Christian Buske

**Affiliations:** 1 Institute of Experimental Cancer Research, CCC and University Hospital Ulm, Germany, 89081 Ulm, Germany; 2 Bing Center for Waldenström’s Macroglobulinemia, Dana-Farber Cancer Institute, Harvard Medical School, Boston, MA USA

**Keywords:** B-cell lymphoma, B-cell lymphoma

## Abstract

It is one of the major aims in cancer research to improve our understanding of the underlying mechanisms which initiate and maintain tumor growth and to translate these findings into novel clinical diagnostic and therapeutic concepts with the ultimate goal to improve patient care. One of the greater success stories in this respect has been Waldenström’s Macroglobulinemia (WM), which is an incurable B-cell neoplasm characterized by serum monoclonal immunoglobulin M (IgM) and clonal lymphoplasmacytic cells infiltrating the bone marrow. Recent years have succeeded to describe the molecular landscape of WM in detail, highlighting two recurrently mutated genes, the *MYD88* and the *CXCR4* genes: *MYD88* with an almost constant and recurrent point mutation present in over 90% of patients and *CXCR4* with over 40 different mutations in the coding region affecting up to 40% of patients. Intriguingly, both mutations are activating mutations leading in the case of CXCR4 to an indelible activation and perpetual signaling of the chemokine receptor. These data have shed light on the essential role of CXCR4 in this disease and have paved the way to use these findings for predicting treatment response to the Bruton tyrosine kinase (BTK) inhibitor ibrutinib and novel therapeutic approaches in WM, which might be transferable to other related CXCR4 positive diseases. Well known for its central role in cancer progression and distribution, CXCR4 is highlighted in this review with regard to its biology, prognostic and predictive relevance and therapeutic implications in WM.

## CXCR4—a key member of the chemokine receptor family in hematopoiesis and cancerogenesis

### Structure and ligands

C-X-C chemokine receptor type 4 (CXCR4), characterized by one amino acid between the two cysteine residues, also named as cluster of differentiation 184 (CD184), is a classical G protein coupled receptor (GPCRs) with its encoded nucleotide sequence located on chromosome 2 [1]. It belongs to the Rhodopsin-like class A of GPCRs, which are membrane proteins of a superfamily that transmit signals from the outside of the cell to activate a plethora of inner molecular pathways. CXCR4 acts as a conventional chemokine receptor. Chemokine receptors are organized into four different groups, depending on the chemokine they bind to and the count and arrangement of the N-terminal cysteine residues i.e. CXC, CX3C, CC, and XC [2]. The surface receptor CXCR4 is ubiquitously expressed and evolutionary conserved with a similarity of 89% between human and mouse orthologues. The surface protein CXCR4 comprising 352 amino acids consists of seven transmembrane helices coiling through the cell membrane of which helix VII is distinctive for CXCR4 compared to other GPCRs. Its uniqueness allows a chemical bond between helix VII and the N-terminal region that belongs to a complex, enabling the binding to CXCL12, the natural ligand of CXCR4 [3].

CXCL12 (stromal cell-derived factor-1, SDF-1), is the natural binding partner of CXCR4 and belongs to a large family of chemokines. The molecular structure of CXCL12 is characterized by a three-stranded β-sheet that is packed against an α-helix [4]. It is ubiquitously expressed in all kinds of tissue with six reported isoforms in human. The chemokine is highly conserved between mouse and human (>92%), suggesting an important role in evolution and has been mapped to chromosome 10q [5]. Within the hematological system, CXCL12 is secreted by stromal cells in the perivascular region, including endothelial cells and mesenchymal progenitors comprising a distinct niche that support HSCs [6]. When genetically deleted, mice have a hypocellular marrow characterized by deficient B-lymphopoiesis and myelopoiesis and abnormal neuronal and cardiovascular development with lethal outcome [7].

CXCL12 binding to CXCR4 initiates a wide network of signal transduction by activating subunit Gα of the heterotrimeric G protein. By switching from GDP to GTP the α unit dissociates from the trimeric structure and binds to GDP. Both, the GTPα and the Gβ/Gγ dimer interact with different effector proteins and initiate various intracellular signaling cascades. The Gα family acts via PLC, to hydrolyze PIP2 and generates two-second messengers, IP3 and DAG. IP3 and DAG mobilize Ca2+ from intracellular stores and are able to activate a number of protein kinases, including PKC, controlling migration and proliferation. Further stimulation leads to the activation of mitogen-activated protein kinase (MAPK) originally called ERK (Extracellular signal–regulated kinases) as well as small GTPases RAS and RHO which are involved in chemotaxis and cell cycle regulation. The main axis PI3K is linked to lymphocyte trafficking, chemotaxis, and cell survival via AKT but can also result in the phosphorylation of several adhesion regulators such as proline-rich kinase-2 (Pyk-2), Crk-associated substrate (CRK), focal adhesion kinase (FAK), and paxillin. The PI3K-AKT-NF-κB axis and also the MEK1/2 and ERK 1/2 pathways are involved in activation and phosphorylation of cellular proteins and transcriptions factors to regulate genes responsible for proliferation, migration, and stemness.

**Fig. 1:**
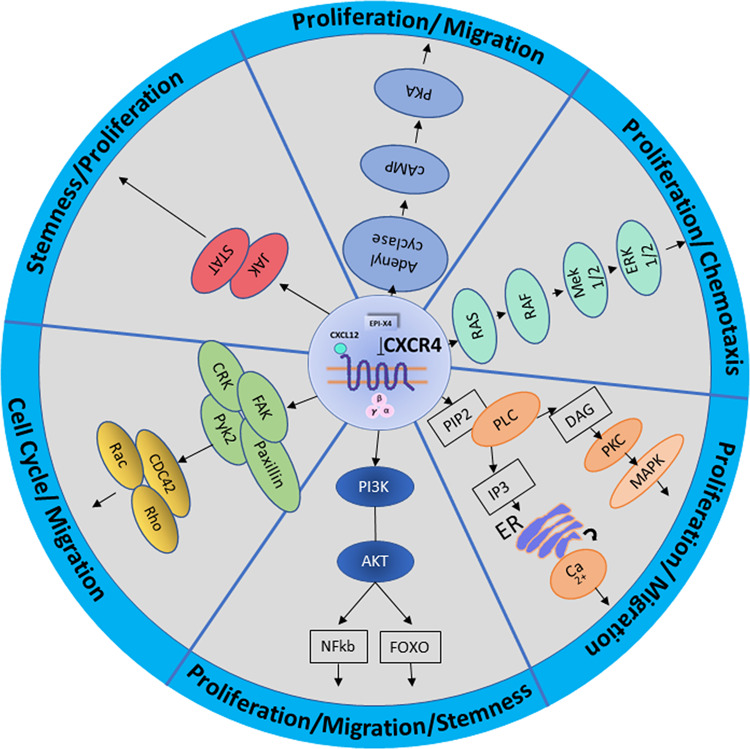
Schematic overview of CXCR4 signaling. Pathways involved in CXCR4 CXCL12/CXCR4 signaling pathways. AKT protein kinase B, PI3K phosphatidylinositol 3-kinase, NFkB nuclear factor kappa-light-chain-enhancer of activated B cells, FOXO forkhead box, FAK focal adhesion kinase, Pyk-2 proline-rich kinase-2, CRK Adapter molecule crk, Rho Ras homolog gene family, Rac Ras-related C3 botulinum toxin substrate, CDC42 cell division control protein 42 homolog, JAK janus kinase, STATsignal transducer and activator of transcription, cAMP cyclic adenosine monophosphate, PKA protein kinase A, Ras rat sarcoma protein family, RAF proto-oncogene serine/threonine-protein kinase, MEK dual specificity mitogen-activated protein kinase, ERK extracellular-signal-regulated kinase, PIP2 phosphatidylinositol bisphosphate, PLC phospholipase C, PKC protein kinase C, IP_3_ inositol trisphosphate, DAG diacylglycerol, ER endoplasmic reticulum, MAPK mitogen-activated protein kinase, Ca^2+^ Calcium ions, CXCR4 C-X-C chemokine receptor type 4, CXCL12 C-X-C chemokine ligand 12.

The signaling is tightly regulated and stopped by internalization processes and lysosomal degradation via arrestin which binds to the phosphorylated receptor and initiates degradation [8] (Fig. 1).

Despite CXCL12, other ligands of CXCR4 have been identified, namely ubiquitin [9], the anti-HIV-1 chemokine vMIP-II [10], the HIV protein gp120 [11] and more recently the endogenous CXCR4 antagonist, EPI-X4, an active serum albumin fragment [12].

### Functional Role in normal and malignant tissue

Its crucial role as factor in directed migration and leukocyte trafficking in the immune system of higher organisms as well as homing of stem and progenitor cells has drawn key attention to CXCR4 in the recent years. The role of CXCR4 starts during earliest stages of development at the 4-cell stage in blastocyst formation, where CXCR4 is expressed and gradually increases in expression during embryo development. This increase of CXCR4 has been shown to have a direct effect on the invasion capability and successful implantation of the human blastocyst [13]. Necessity of CXCR4 for embryonic viability has been shown in mice, as knock-out mice are embryonic lethal with defects in vascular development, hematopoiesis, and cardiogenesis [14]. In later development stages it is involved in organ formation with concise tissue-specific functions for example as leading signal for axon solicitation in neural tissue [15]. Within the hematopoietic system CXCR4 is expressed on most hematopoietic cell types including lymphocytes, stromal fibroblasts, endothelial and epithelial cells as well as hematopoietic stem cells and cancer cells [16]. In the adult BM CXCR4 controls stem cell retention in the BM and its disruption leads to a release of the HSC pool with the ability to restore normal blood cell population or to maintain homeostasis with enhanced release on demand during infections and injuries. Recently, CXCR4 signaling has been found to play a fundamental role in restoring the HSC pool and reconditioning the microenvironment after stem cell transplantation via upregulating the antiapoptotic protein Survivin [17]. These aspects of the role of CXCR4 have been described in more detail in excellent reviews [15, 18]

CXCR4 has been associated with cancerogenesis for a long time: elevated CXCR4 expression levels have been portrayed in numerous cancers with relation to poor prognosis and therapy resistance [19]. Upregulation was linked to several factors, such as hypoxia-inducible factor-1 (HIF-1) [20] as well as several growths factors such as fibroblast growth factor FGF [21], vascular endothelial growth factor (VEGF) [20] and hepatocyte growth factor (HGF) [22]. Also transcription factors like nuclear respiratory factor-1 positively influence CXCR4 expression [23] whereas Yin-Yang 1 (YY1) negatively regulates the promoter activity of the receptor [24]. Furthermore, the macrophage migration inhibitory factor MIF has been associated with tumor aggressiveness and metastatic spread. Upon binding to CXCR4, the MIF/CXCR4 complex is involved in regulation of endothelial progenitor cell migration, metastasis, cancer growth [25] and homing to tumors [26]. However, the implication of CXCR4 with tumorigenesis relies largely on expression studies and functional assays proving direct oncogenicity of CXCR4 in solid cancers is rare.

In hematological malignancies high expression of CXCR4 is observed in several entities ranging from acute leukemias such as acute lymphoblastic leukemia (ALL) and acute myeloid leukemia (AML) to a variety of malignant lymphomas including chronic lymphocytic leukemia (CLL), diffuse large B cell lymphoma (DLBCL), follicular lymphoma (FL), marginal zone lymphoma (MZL), multiple myeloma (MM), hairy cell leukemia (HCL) and mantle cell lymphoma (MCL) [27–32]. Furthermore, detection of the phosphorylated form of CXCR4, pCXCR4, is an indicator for poor survival in adult patients with B-ALL [33]. Furthermore, high expression of CXCR4 is a hallmark of CLL cells compared to normal B cells [34]. Ghobrial et al. saw a significant increase in the expression of CXCR4 in B-CLL cells from patients with RAI stage IV compared with stage 0 [35]. A large multi-center study described CD49d as an independent prognostic marker in CLL and suggested that CD49d and CXCR4 may be up regulated in a co-ordinated fashion and linked to poor prognosis prediction [36].

## Waldenström’s Macroglobulinemia—a scylla like heterogenous lymphoma

### Clinical characteristics

76 years have passed since Jan Waldenström reported about a disease, named later Waldenström’s Macroglobulinemia that was characterized besides salient clinical features like fatigue, epistaxis, and lymphadenopathy by bone marrow lymphocytosis and an increased amount of serum macroglobulin [37]. Already a few years earlier Bing and Neel had reported about three cases with macroglobulinemia and central nervous affection, these cases are designated as Bing–Neel syndrome [38, 39]. Since then tremendous progress has been made in characterizing the underlying biology and genetic landscape in WM, but still today WM is defined as clinicopathological entity characterized by the presence of a lymphoplasmacytic lymphoma in the bone marrow (BM) and an elevated monoclonal serum IgM level [40, 41]. The cellular composition of WM comprises malignant lymphocytes and plasma cells, forming a picture of a scylla—like heterogenous hybrid disease. Its clinical picture is accordingly heterogenous characterized by constitutional symptoms, anemia, fatigue as well as IgM related symptoms such as hyperviscosity or neuropathy [42, 43]. WM is rare, with an incidence rate of 4–7 per 1 million European women and men per year, respectively [44], presenting 1–2% of all hematological disorders. It is a disease of the elderly and occurs more frequently in male than in female and in Caucasians than in African Americans [45]. WM is incurable and its cause is unknown, however, evidence for a high familial incidence is compelling and approximately one-fourth of patients have family members with a history of lymphoproliferative neoplasias [46]. WM is often originating from IgM-monoclonal gammopathy of undetermined significance (MGUS) [47] and transformation into aggressive diffuse large B‐cell lymphoma (DLBCL) [48] as well as leukemia [49] has been reported. WM has an indolent clinical cause in the majority of cases and mortality varies in patients with WM. Overall survival rates (OS) at 10 years are around 40% with a median age of 69 at diagnosis. In another analysis the relative survival (taking the expected survival of the general population in a specific calendar year into account) was 57%, based on the Surveillance, Epidemiology, and End Results (SEER) database and including patients with WM diagnosed between 1980 and 2010 (*n* = 7744) [50, 51]. However longer survival rates have been observed in patients <45 with 10‐year OS rates of 86% [52]. Main causes of death being progression, transformation to a high-grade lymphoma, or therapy-related complications [50].

### Genetic landscape

Since Burnet who postulated genetic factors in the form of mutations might be basis of neoplasms with abnormal paraprotein production, many genetic abnormalities have been linked to WM [53]. In the 70ties Spencer et al. remarkably illustrated his hypothesis in a case study with twins of which one twin acquired WM whereas the other was healthy, the one affected individual showing an acquisition of an extra Chromosome A, linking this chromosomal gain to WM [54]. A few years later this finding was confirmed and found in 3 additional patients [55], therefore emphasizing that a hereditary pre-disposition might be an important contributing factor in this disease. Today’s evidence for a familial predisposition in WM is striking mapping 26% of WM patients to family members with either WM or another B-cell disorder in a large single-center study evaluating 924 cases [56].

Beside an obvious familial disposition first array-based genomic hybridization analysis demonstrated that 83% of the WM patients carry chromosomal abnormalities. Chromosome 6q 21–25 deletions are the most frequent observed aberrations, affecting 30% of patients [57], whereas gains in 6p are observed in every 5^th^ patient and are mainly described as a subsequent event of 6q deletions [58]. Further cytogenetic disorders include 13q14 and 17p deletions as well as trisomy 4, 12, and 18 [59]. Research also has directed attention towards the role of microRNAs in the biology of WM as well as to the impact of epigenetic alterations such as histone acetylation downregulating microRNAs such as miRNA-9* [60]. An increased expression has been identified for miRNAs-363*, −206, −494, −155, −184, and −542–3p of which mi-RNA-155 was further validated and proved as growth-promoting oncogenic factor in the WM cell line BCWM.1 [61].

### The CXCR4 mutation—a major driver in WM biology

The most recent and striking development in WM genetics lead to the identification of highly recurrent somatic mutations in two genes, namely MYD88 and CXCR4, which clearly has paved the way to a deepened understanding of the signaling cascades driving growth and importantly also therapeutic resistance in WM [62].

MYD88 mutations occur mainly monoallelic in over 90% of patients with WM and with this are by far most common though not specific for this lymphoma subtype [63]. They also occur at very low frequencies in diffuse large B-cell lymphoma (DLBCL), marginal zone lymphoma (MZL), and chronic lymphocytic leukemia (CLL), but allow differentiation to other lymphomas such as IgM multiple myeloma (MM), which virtually never shows MYD88 mutations [64]. The vast majority of mutations change the T by C at position 265 (MYD88 point mutation L265P), but alternate mutations occur as subclonal event in C to G resulting in an amino acid substitution (S219C) (1 patient) or alternative mutations M232T and S243N [58, 59]. A so far unknown mutation was recently described to cause the substitution of L265 with RPP (L265RPP). MYD88 is a protein adapter that contains three main domains including a death domain (DD), an intermediate linker domain (ID), and the toll/interleukin-1 receptor domain (TIR) at the C terminus. Upon recruitment from Toll-like receptor (TLR), MYD88 activates the IL-1 receptor signaling pathway via interleukin-1 receptor-associated kinases 1, 2, and 4 (IRAK1, IRAK2, IRAK4) leading to a phosphorylation and constitutive activation of BTK (Bruton’s tyrosine kinase). BTK, a critical kinase in B-cell receptor (BCR) signaling that regulates immune response, cell proliferation, and cell death through activation of the NF-KB signaling pathway (p50–p65) and the mitogen-activated protein kinase (MAPK) pathway. BTK interacts with four different proteins downstream of TLR signaling including TIR, MYD88, IRAK1, and TIRAP/MAL (TIR domain containing adapter protein TIRAP or MyD88 adapter-like (MAL)). In WM the level of activated BTK is higher when MYD88 is mutated compared to the unmutated cells. The biology of MYD88 mutations has been reviewed in depth elsewhere [65–67].

The second most recurrent mutation in WM hits the CXCR4 gene: mutations in this gene can be found in up to 40% of the patients. In total more than 40 mutations were described so far [68], all exclusively found in the regulatory cytosolic domain stretching from amino acid position 308 to 352. Unlike MYD88, multiple CXCR4 mutations can be present in an individual appearing with heterozygous character in different clones [59].

**Fig. 2:**
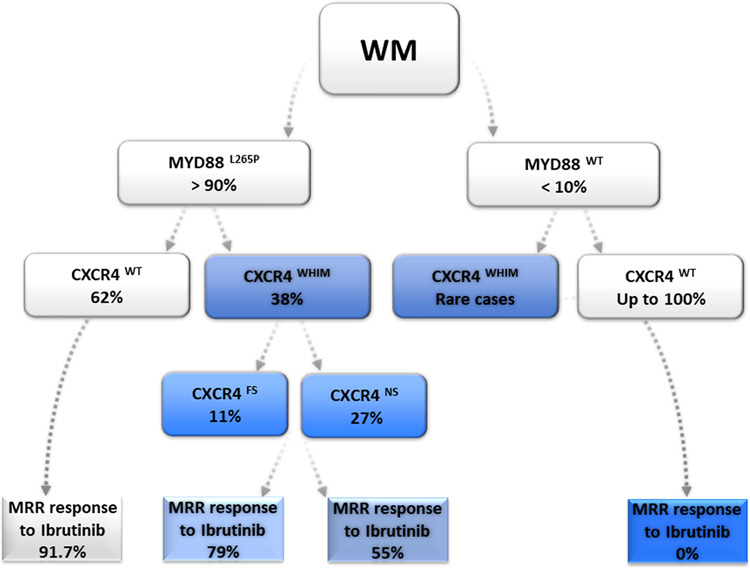
MYD88 and CXCR4 mutations in WM. Summary of the distribution of MYD88 and CXCR4 mutations in WM (according to [58, 68, 76, 77]). MRR are given for Ibrutinib (for MYD88^L265P^ / CXCR4^WT^, MYD88^WT^ / CXCR4^WT^ according to [76, 77] (*n* = 62) and for CXCR4 FS (*n* = 49) versus NS (*n* = 19) according to [68]) NS nonsense, FS frameshift, WT wildtype WHIM Warts, Hypogammaglobulinemia, recurrent bacterial Infections and Myelokathexis, MRR major responds rate.

The most frequent (50%) mutated region is the amino acid S338X at position 1013 with nucleotide changes C > G in 54% and C > A in 25% of the cases, both resulting in a stop codon, followed by S338 frameshift mutation in 21% of the cases. Almost all mutations described in WM introduce a premature stop codon or a frameshift that impair CXCR4 desensitization and internalization, thereby prolonging signaling upon binding of the chemokine ligand CXCL12 (Fig. 2) [58, 69].

The main signaling axis that is affected by CXCR4 mutations promote enhanced AKT and subsequent MAPK 1/2 signaling, resulting in sustained survival signals for cancer cells [70]. Perpetual signaling enhanced by nonsense as well as frameshift mutations have been investigated with regard to their function in WM. Stimulation of engineered WM cells with CXCL12 resulted in significantly reduced levels of CXCR4 receptor internalization compared to the wildtype control. Both frameshift and nonsense mutation potentiate MAPK signaling following CXCL12 stimulation when compared to the wildtype. Direct comparison of frameshift versus nonsense CXCR4 mutations reveal an enhanced MAPK signaling for the nonsense mutation S338X. The results confirm clinical observations of CXCR4 S338X showing prolonged MAPK signaling compared to frameshift mutations and present with higher tumor burden. The reason for the variation of MAPK1 activation is still in abeyance but possible explanations might be the loss of CXCR4 C‐terminal binding sites for β‐arrestin that sustains MAPK1 signaling or a difference in CXCR4 dimer formation and subsequent downstream signaling. When testing sensitivity to ibrutinib, CXCR4 mutant‐expressing cells were rescued by CXCL12 from ibrutinib‐induced apoptosis and the effect could be abrogated by adding plerixafor [71]. By determining the cancer cell fraction (CCF) of CXCR4 mutations in WM the mutation S338X was identified as mostly sub-clonal with variable clonal distribution, which points to the mutation as a late oncogenic event acquired after MYD88 mutation. The analysis was performed using parallel quantitative AS‐PCR analyses for MYD88 L265P and CXCR4 S338X from 21 untreated WM patients resulting in a highly clonal distribution. With 13 patients the majority expressed CXCR4 S338X C > G only and when analyzed in relation to MYD88 L265P showed a clonal distribution of 44.5%. For 7 WM patients who expressed both CXCR4 S338X mutations (C > G and C > A), the fraction of cells expressing CXCR4 S338X relative to MYD88 L265P was around 4% [72, 73]. In contrast to data on CXCR4 mutations data on the expression levels of CXCR4 are scarce in WM compared to normal counterparts. However, it could be demonstrated that transcription of CXCR4 is high in WM particularly in patients with MYD88^MUT^/CXCR4^WT^ compared to circulating normal B cells [74].

### Impact of CXCR4 mutations on treatment outcome in WM patients

Patients with CXCR4 mutations are confronted with the downside of showing higher bone marrow involvement, higher IgM levels, symptomatic hyperviscosity, and a more aggressive disease at diagnosis with reduced sensitivity toward the BTK—inhibitor ibrutinib. Of note, CXCR4 mutations occur already in asymptomatic WM and the presence of these mutations was associated with a shorter treatment-free survival compared to patients without CXCR4 mutation [75].

Data particularly from trials testing the BTK inhibitor ibrutinib have shown that CXCR4 mutations can impact clinical outcome in patients with WM, underlining the key importance of this gene also in the clinical setting. The following sections describe our current knowledge on the influence of the CXCR4 mutational status on therapy outcome in this disease (summarized in Table 1).

**Table 1:** Treatment outcome in WM patients according to the CXCR4 mutational status treated with BTK – inhibitors.

Trial	Treatment	Patient population	MYD88^MT^/CXCR4^WT^			MYD88^MT^/CXCR4^MT^			MYD88^WT^/CXCR4^WT^		
			*MR*	*VGPR*	*PFS*	*MR*	*VGPR*	*PFS*	*MR*	*VGPR*	*PFS*
ClinicalTrials.gov no NCT01614821 (Single arm phase II) [76, 77]	Ibrutinib single agent	Relapsed/refractory WM (*n* = 63)	97%	44%	5-year 71%	64%	9%	5-year 34%	0%	0%	2-year 0%
ClinicalTrials.gov no NCT02604511 (single arm, phase II) [64]	Ibrutinib single agent	Treatment naive WM (*n* = 30)	94%	31%	na	71%	7%	na	na	na	na
*iNNOVATE*; ClinicalTrials.gov no NCT02165397 (randomized Phase III) [108]	Ibrutinib/Rituximab (I/R) vs Placebo/Rituximab	Treatment naive and relapsed WM (*n* = 150)	IR: 94%	IR: 38%	3-year PFS:84%	IR:73%	IR:15%	3-year PFS:64%	IR:64%	IR:27%	3-year PFS:82%
*ASPEN*; ClinicalTrials.gov no NCT03053440 (randomized Phase III MYD88^MT^; single arm observational MYD88^WT^) [80]	Zanubrutinib (Z) vs Ibrutinib (I) (MYD88^MT^) Zanubrutinib (MYD88^WT^)	Treatment naive and relapsed WM (*n* = 201; MYD88^M^) (*n* = 28; MYD88^WT^)	Z:82% I:82% na	Z:34% I: 24% na	na na	Z:70% I:65% na	Z:18% I: 10% na	na na	na Z:50%	na Z:27%	na 1.5 year PFS 68%

*MR* major response, *VGPR* very good partial response, *PFS* progression free survival, *MT* mutated, *WT* wildtype, *Z* zanubrutinib, *I* Ibrutinib.

### BTK inhibitors

**Fig. 3:**
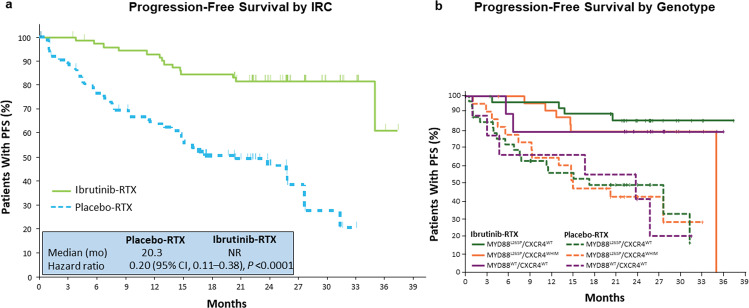
Progression-free survival of WM patients treated with ibrutinib/rituximab versus placebo/rituximab. The iNNOVATE phase III randomized study (**a**) for the total patient group and (**b**) depending on the MYD88/CXCR4 mutational status [108].

Targeting the BTK in WM cells by BTK inhibitors has changed the treatment landscape in WM: in the pivotal phase II study 63 symptomatic relapsed patients with Waldenström’s macroglobulinemia received Ibrutinib at a daily oral dose of 420 mg until disease progression or the development of unacceptable toxicity. This oral single-agent treatment resulted into an overall response rate of 90.5% and a major response rate of 73%, characterizing ibrutinib as the most efficient single-agent treatment in WM. Of note, responses varied with regard to the mutational status of MYD88 and CXCR4 with highest responses in the MYD88^Mut^/CXCR4^WT^, intermediate responses in the MYD88^Mut^/CXCR4^Mut^ and lowest responses in the MYD88^WT^/CXCR4^WT^ cases (Fig. 2). In particular nonsense CXCR4 mutations in contrast to frameshift mutations were associated with delayed time to response, less deep responses, and shorter PFS upon treatment with ibrutinib monotherapy [68]. The estimated 2-year progression-free and overall survival rates among all patients were 69.1% and 95.2%, respectively. Treatment-related toxic effects of grade 2 or higher were similar as observed in other related lymphomas such as chronic lymphocytic leukemia or mantle cell lymphoma and included neutropenia (in 22% of the patients) and thrombocytopenia (in 14%). Postprocedural bleeding and atrial fibrillation associated with a history of arrhythmia occurred in 3% and 5% of cases, respectively [69, 76, 77]. A prospective study for untreated WM patients, in which all 30 patients were MYD88 mutated and 47% had additional CXCR4 mutations, confirmed the results. Overall and major responses for all patients were 100% and 83%, respectively. As seen in relapsed patients responses were depending on the mutational status with a drop in major (94% v 71%) and very good partial (31 v 7%) responses for patients with mutated CXCR4 and delayed time to major responses in this patient group (1.8 v 7.3 months; *P* = 0.01) [64]. Responses to Ibrutinib and also PFS depends also on the allelic burden of CXCR4 as was shown in a smaller retrospective analysis of 37 patients treated with ibrutinib: in patients with CXCR4^S338X^ mutations, clonality of ≥25% was associated with lower response rates and inferior PFS compared to patients with <25% clonality [72]. Based on the observation, that ibrutinib single agent has less activities in CXCR4 mutated patients and in patients with non-mutated MYD88 and CXCR4, a large international prospective study performed on behalf of the European Consortium for Waldenström’s Macroglobulinemia (ECWM) was initiated, randomizing 150 patients with treatment naive or pretreated WM between ibrutinib plus rituximab or placebo plus rituximab. Primary endpoint was PFS, which was significantly superior in the ibrutinib arm at 30 months (82% with ibrutinib–rituximab versus 28% with placebo–rituximab; hazard ratio for progression or death, 0.20; *P* < 0.001) (Fig. 3). Importantly, addition of rituximab to ibrutinib resulted into a largely homogenous response rate and PFS independent of the mutational status of MYD88 and CXCR4, demonstrating that the combination can increase response, time to response, and PFS in WM with double mutated MYD88 and CXCR4 as well as in WM with both genes non-mutated (Fig. 2). Atrial fibrillation and hypertension of grade 3 or higher occurred more frequently with ibrutinib–rituximab than with placebo–rituximab (12% vs. 1% and 13% vs. 4%, respectively); in contrast, infusion reactions and any grade of IgM flare occurred less frequently in the ibrutinib arm (1% vs. 16% and 8% vs. 47%, respectively). Based on these data ibrutinib in combination with rituximab was approved for treatment-naive and relapsed patients with WM by the FDA and the EMA [78].

Second generation BTK inhibitors are characterized by less off target effects and aim at increasing efficacy and reducing side effects seen with ibrutinib. More recently, a randomized Phase II trial reported excellent results in 102 patients with treatment naive or relapsed WM with single-agent acalabrutinib, given 100 mg twice a day until progression or non-tolerated toxicity. After a median follow-up of 27·4 months response rate was 93% for treatment naive patients and 93% for relapsed/refractory patients. There seemed to be less atrial fibrillation compared historically to ibrutinib. Data on the impact of CXCR4 mutations were preliminary in this study as there was no central laboratory assessment of CXCR4 mutations in this study [79]. Zanubrutinib, another second generation BTK inhibitor has shown encouraging data in CLL and in a limited number of WM patients and was recently tested in a randomized phase III trial in MYD88 mutated patients in a head-to-head comparison with single-agent ibrutinib. Responses were deeper with Zanubrutinib, but did not translate into a longer PFS or OS in this study, although the follow-up at the time of presentation was still short with 19.4 months. A post-hoc analysis of baseline bone marrow from 190 (95%) patients using next generation sequencing (NGS) for CXCR4 mutation detection revealed the presence of CXCR4^WHIM^ mutations in 53 (28%) of all patients. VGPR rates demonstrated a higher rate overall of 29% versus 18% for CXCR4^WT^ versus CXCR4^WHIM^ patients, a trend also seen in the ibrutinib arm [80]. Longer follow-up of the aforementioned study is needed to confirm whether these new BTK inhibitors are indeed more efficient and/or less toxic compared to ibrutinib in WM. Taken together, also in CXCR4 mutated patients BTKi stays an important backbone of treatment, but addition of Rituximab to ibrutinib or a second-generation BTKi should be considered in these patients, in particular in the case of delayed and/or insufficient response.

### Proteasome Inhibitors

Among the proteasome inhibitors bortezomib is widely used for the treatment of WM based on several phase II trials demonstrating efficacy of bortezomib used as a single agent or in combination with rituximab in WM [81–83]. Intriguingly, so far retrospective data suggest that Bortezomib acts independent of the MYD88 and CXCR4 mutational status: in a retrospective analysis of a phase II study comprising 63 treatment naive or relapsed patients treated with bortezomib/rituximab (bortezomib IV weekly at 1.6 mg/m^2^ on days 1, 8, and 15, every 28 days 3 6 cycles, and rituximab 375 mg/m^2^ at days 1, 8, 15, and 22, on cycles 1) PFS and OS was independent of the CXCR4 mutational status in MYD88 mutated patients. In this retrospective analysis 43 patients were evaluable for CXCR4 mutations with 17 patients being CXCR4 mutated. All CXCR4 mutated patients carried also the MYD88 L265P mutation. Thus, this study did not allow to test the efficacy in MYD88 non-mutated WM [84]. Recent data of a phase I/II study of the ECWM using the orally applicable proteasome inhibitor ixazomib in combination with dexamethasone and rituximab also induced comparable response rates and PFS in CXCR4 mutated versus wildtype cases, although the patient number was limited with 59 patients evaluable in total and 88% of patients with a MYD88L265P mutation and 35% with a CXCR4 mutation [85]. In a recently published study, 26 treatment naïve WM patients received a combination of ixazomib, dexamethasone and rituximab (IDR) for 6 monthly induction cycles followed by 6 every-2-month maintenance cycles. All the patients carried a *MYD88 L265P* mutation and from these 58% a *CXCR4* mutation. Positivity for the CXCR4 mutation delayed the median time to response (TTR) and time to major response (TTMR) (median TTR of 3 and 1 month, respectively (*p* = 0.003), and median TTMR of 10 and 3 months, respectively (*p* = 0.31)). Also the rate of VGPR were lower in CXCR4 mutated patients (with and without *CXCR4* mutations 7% and 36%, respectively (*p* = 0.06)), whereas the median PFS, duration of response and time to next treatment was not affected [86]. Another proteasome inhibitor sparing neurotoxicity associated with bortezomib is carfilzomib. Carfilzomib was evaluated in combination with rituximab and dexamethasone (CaRD) in WM patients that were naive to both Bortezomib and rituximab [87]. CaRD therapy consisted of IV Carfilzomib given at 20 mg/m^2^ (on cycle 1), and on 36 mg/m^2^ thereafter (cycles 2–6), on days 1, 2 and 8, 9 with dexamethasone 20 mg on days 1, 2, 8, 9 and rituximab 375 mg/m^2^ on days 2, 9 every 21 days. Maintenance therapy followed 8 weeks after cycle 6 with IV Carfilzomib 36 mg/m^2^ and dexamethasone 20 mg on days 1, 2 and rituximab 375 mg/m^2^ on day 2 every 8 weeks for 8 cycles. The overall response rate in this study was 87%, and 68% of the patients achieved a major response. In this study, MYD88 and CXCR4 tumor mutational status were examined and did not appear to impact response attainment, although the patient numbers are small. The impact of carfilzomib in combination with ibrutinib will be tested in a large international phase III study of the ECWM, randomizing treatment naive or relapsed patients molecularly profiled for MYD88 and CXCR4 mutations between carfilzomib/ibrutinib versus ibrutinib (Clinicaltrials.gov No. NCT04263480).

### Rituximab/chemotherapy

Data on the impact of CXCR4 mutations on the outcome of rituximab/chemotherapy are scarce: in a multi-center retrospective study performed between January 2013 and December 2017 in France 69 patients were enrolled. 45 of 51 (88%) and 11 of 44 tested patients (25%) were mutated for MYD88 and CXCR4, respectively. The regimen induced an overall (ORR) of 97% and a major (MRR) response of 96%. Thirteen (19%) patients achieved a complete response (CR), 26 (37%) very good partial response (VGPR), 28 (40%) partial response (PR) and 1 (1%) MR; one patient had stable disease. Of note, disease response was not different between patient with or without CXCR4 mutation [88].

### Targeting CXCR4 as an innovative therapeutic approach

Based on the role of CXCR4 as an oncogenic factor in a variety of cancers, targeting of CXCR4 has been recognized as a highly attractive therapeutic principle since many years. Initially, CXCR4 antagonists were identified and developed for HIV treatment as CXCR4 acts as a co-factor for cell entry of the virus. CXCR4 antagonists can be separated into four groups: peptides, such as EPI-X-4 or T140, non-peptides such as the clinical approved biclam AMD3100, antibodies against CXCR4 and optimized agonists and antagonist [89].

**Fig. 4:**
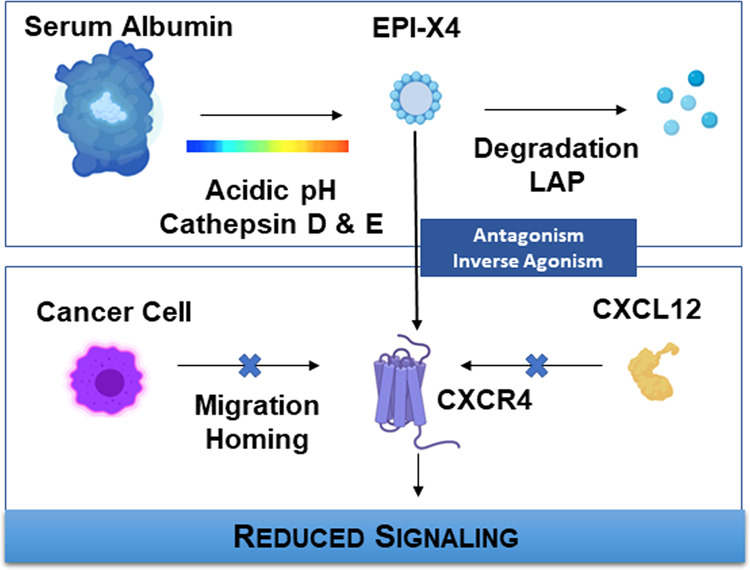
Summary of the generation, interaction, and function of the CXCR4 antagonist EPI-X4. The endogenous peptide EPI-X4 is cleaved from highly abundant serum albumin under low pH conditions. The generation is controlled by aspartatic proteases Cathepsin D and E and stopped by leucylaminpeptidases (LAPs) within a relatively short time-frame. After its release EPI-X4 binds to CXCR4 in a very specific manner and competes with CXCL12 binding, acting as an antagonist. Its inverse agonist ability furthermore suppresses constitutive signaling activity of CXCR4 in the absence of CXCL12. By cordoning off the interaction between CXCR4 and its agonist CXCL12, EPI-X4 mobilizes hematopoietic stem cells and is able to suppress migration and homing of cancer cells.

Peptides and peptide derivatives have been used first to develop CXCR4 antagonists. Initially, the group of polyphemusin peptides was discovered screening naturally occurring peptides from horseshoe crabs for HIV entry blocking capacity [89]. Chemical modification has led to BKT140 also known as BL-8040 (TN14003) which is an analog of T140 and in focus of recent pre-clinical and clinical trials in patients with leukemia and lymphoma [89] (Table 2). Recent research has focused on exploring peptide libraries to identify promising bioactive peptides from human sources [90, 91]. Screening of the hemofiltrate had been successful in 2015 when Zirafi and colleagues identified a, so far unknown highly specific CXCR4 antagonist named EPI-X4. The 16-mer peptide EPI-X4 is a segment of albumin and results from resecting Cathepsins under acidic conditions which is a characteristic of inflammation. Cathepsins are secreted from nearly all cell types and it is known that Cathepsin D expression is high in B cell lymphomas [92]. In contrast to AMD3100 EPI-X4 does not bind CXCR7 and has no mitochondrial cytotoxicity, suggesting lower side effects. Intense research has already shown that EPI-X4 is able to interfere in the crosstalk between CXCR4 and CXCL12, inhibits HIV-1 infection, mobilizes hematopoietic stem cells and is able to suppress migration of leukemic cells in vitro [12] (Fig. 4).

**Table 2:** CXCR4 antagonists and antibodies under clinical investigation (source ClinicalTrials.gov).

Drug	Trial phase	Status	Condition	ClinicalTrials.gov identifier
BKT140	Phase I/II	Completed	Multiple Myeloma	NCT01010880
BMS-936564 Lenalidomide Dexamethasone Bortezomib	Phase I	Completed	Multiple Myeloma	NCT01359657
Ulocuplumab Lenalidomide Dexamethasone Bortezomib	Phase Ib/II	Completed	Multiple Myeloma	NCT01359657
Ulocuplumab Ibrutinib	Phase I/II	Active	WM	NCT03225716
Mavorixafor Ibrutinib	Phase I	Recruiting	WM	NCT04274738
BMS-936564	Phase I	Completed	AML, DLBCL, CLL FL	NCT01120457
Plerixafor		Recruiting	MM	NCT03406091
BL-8040 (BKT-140) Nelarabine	Phase II	Recruiting	T-ALL	NCT02763384
TG-0054	Phase I	Completed	Healthy	NCT00822341
Plerixafor	Phase II	Completed	Healthy	NCT00075335
Plerixafor	Prospective observational	Completed	Lymphoma Myeloma	NCT01700608
Plerixafor G-CSF	Phase II/III	Active, not recruiting	WHIM	NCT02231879
Plerixafor	Phase I/II	Recruiting	WHIM	NCT00967785
Plerixafor Mitoxantrone Etoposide Cytarabin	Phase I/II	Completed	AML	NCT00512252
Plerixafor	Phase I/II	Completed	AML	NCT01141543
POL6326	Phase I	Completed	Healthy	NCT01841476
BL-8040 Ara-C	Phase II	Completed	AML	NCT01838395

Bicyclams belong to the non-peptidic group of small molecules that target CXCR4. Within this group AMD3100 represents the most prominent and known molecule which got approved by the FDA in 2008 for mobilization of hematopoietic stem cells of non-Hodgkin lymphoma and multiple myeloma [93]. Another approach to target CXCR4 is the development of antibodies directed against CXCR4. Here, BMS-936564 / MDX1338 also called Ulocuplumab belongs to the most promising candidates and is currently under investigation in clinical phase I, including a phase Ib/II trial in multiple myeloma, combining the anti-CXCR4 antibody with lenalidomide or bortezomib plus dexamethasone, showing encouraging clinical activity [94, 95]. A selection of CXR4 antagonists and antibodies and their stage of development in terms of hematologic neoplasms can be found in Table 2.

In WM the appealing approach to counteract constitutive activation mutated CXCR4 by CXCR4 antagonists has just begun to be tested in clinical trials. Results from a prospective phase I/II study evaluating ibrutinib in combination with the anti-CXCR4 monoclonal antibody ulocuplumab in WM patients who carry CXCR4 mutations, are awaited soon (NCT03225716).

Ulocuplumab inhibits the binding of CXCR4 to CXCL12, and has shown both in vitro as well as in vivo anti-tumor in a WM tumor xenograft model engrafted with CXCR4^WHIM^ mutated tumor cells [96]. Ulocuplumab demonstrates pharmacokinetics which allows weekly induction, and bi-weekly maintenance therapy, which stands in contrast to the short half-life of plerixafor [97]. Based on these data a prospective Phase I/II clinical study of ulocuplumab with ibrutinib was started for WM patients with CXCR4 mutation using three different dose cohorts with the goal to improve ibrutinib responses in CXCR4 mutated patients. The same concept follows a just recently activated phase 1b multi-center, open-label, dose-escalation trial assessing the safety and tolerability of mavorixafor in combination with ibrutinib in MYD88 mutated WM patients harboring an additional CXCR4 mutation (NCT04274738). Changes in serum immunoglobulin M (IgM) and hemoglobin (Hgb) from baseline will be used as biomarkers of clinical response. The clinical trial is expected to enroll ~12–18 patients and is conducted as part of a collaboration with The Leukemia & Lymphoma Society to accelerate the development of mavorixafor for the treatment of WM. Mavorixafor (AMD070) is a potent, selective, and orally bioavailable CXCR4 chemokine receptor allosteric antagonist with an IC50 value of 13 nM against CXCR4 125I-SDF binding [98].

### Future developments

WM is an example, which demonstrates that insights into the biology has been successfully translated into clinical concepts as shown for the class of BTK inhibitors as well as for CXCR4 inhibitors. As we can state already that BTK inhibitors have changed the treatment landscape in WM, it is too early to say this for the CXCR4 inhibitors. But first data are indeed encouraging and a success of this class of compounds in WM would be another excellent proof that understanding the biology of WM is the key which opens the doors to innovative treatments. CXCR4 inhibitors will be not the end of the road. There were early data on BCL-2 expression in WM independently of the genotype and first data have shown promising activity of the BCL-2 inhibitor venetoclax in relapsed/refractory MYD88 and CXCR4 mutated patients with WM [99, 100]. In addition, WM comprises a cellular compartment with plasmacytic differentiation, being responsible for the IgM production of the malignant clone. These cells are positive for CD38 and trials are ongoing testing daratumumab in patients with WM. Another fascinating and greatly promising novel approach is the CAR-T cell therapy, which has shown highly promising activity in lymphoma subtypes such as DLBC and MCL [101–103]. Data in indolent lymphoma are still limited with first encouraging data reported for follicular lymphoma [104]. In a case report a patient with transformed WM was successfully treated with CAR-T cells, notably also with eradication of the indolent lymphoma residues [105]. Beside these implications for treatment the MYD88 and CXCR4 mutational status can also guide diagnostics: thus, it is known that MYD88 mutations can help to discriminate WM from IgM positive MM, as the latter one is virtually in all cases MYD88^WT^ [106]. In addition, presence of the CXCR4 mutation can be indicative for WM, but is not specific and was described in in rare cases of DLBCL prolymphocytic leukemia and follicular lymphoma [107] (https://dcc.icgc.org/). All this exemplifies that the future in WM looks bright based on a dynamic research community which drives translational research and by this paves the way to a better clinical management of WM patients.
